# Epidemiologic characteristics of high-risk HPV and the correlation between multiple infections and cervical lesions

**DOI:** 10.1186/s12879-023-08634-w

**Published:** 2023-10-07

**Authors:** Qinli Luo, Xianghua Zeng, Hanyi Luo, Ling Pan, Ying Huang, Haiyan Zhang, Na Han

**Affiliations:** Chongqing Cancer Multi-Omics Big Data Application Engineering Research Center,, Chongqing University Cancer Hospital, 181 Hanyu Road, Shapingba District, Chongqing, 400030 China

**Keywords:** Human papillomavirus (HPV), Genotype, ThinPrep cytological test, Multiple infections

## Abstract

**Background:**

The aim of this study was to determine the prevalence of high-risk human papillomavirus (HR-HPV) and the correlation between multiple infections and cervical lesions.

**Methods:**

The current study involved population-based sample of 20,059 women who underwent cervical screening for 15 HR-HPV genotypes with ThinPrep cytologic test (TCT) results. The correlation between multiple HPV genotype infections and cervical lesions was also determined. The odds ratios (ORs) were calculated to assess co-infection patterns for each genotype with 15 other genotypes and the additive statistical interactions were evaluated.

**Results:**

There was a bimodal pattern among multiple HPV infections, with a peak in the younger group and a second peak in the elderly group. Indeed, most multiple HPV genotypes exhibited a bimodal pattern. The most common HPV type in patients with high-grade squamous intraepithelial lesions (HSILs) was HPV-16, followed by HPV-52, HPV-58, and HPV-33. The most frequent HPV type in patients with cervical cancer was HPV-16, followed by HPV-58 and HPV-33. Women with multiple infections were at a increased risk of low-grade squamous intraepithelial lesions [LSIL] (OR = 2.01; 95% CI 1.38–2.93) and HSIL (OR 2.28; 95% CI 1.36–3.81) when compared to women with single infections. patients with cervical cancer had the higher percentage of multiple HPV infections. Based on the data herein, we suggest that HPV-33 and HPV-58 may also be high-risk HPV types worthy of increased surveillance and follow-up. Conclusion: Our findings suggested that the association between multiple HPV infections and HSIL and LSIL are stronger compared to single HPV infections. There may be some specific combinations that synergistically affected the risk of HSIL and LSIL.

## Novelty & impact statements

Our findings suggested that the association between multiple HPV infections and HSIL and LSIL are stronger compared to single HPV infections. There may be some specific combinations that synergistically affected the risk of HSIL and LSIL.

## Introduction

Human papillomavirus (HPV) infection is the primary cause of cervical cancer [1]. Of cervical cancer cases, 99% are due to high‐risk HPV (HR‐HPV) infections [2]. To date, > 200HPV genotypes have been identified [3]. The American Cancer Society (ACS) recommends that HPV screening alone the preferred method for women 25–65 years of age [4]. Therefore, HPV screening is very important for cervical cancer prevention and detection. The HPV subtypes have different cervical carcinogenicities. Although HPV-16 and HPV-18 account for approximately 70% of invasive cervical cancers worldwide [5], other HR‐HPV genotypes can also cause cervical cancer. It has been recently reported that HPV-16 and HPV-33 are the most common single HR-HPV genotypes in patients with cervical intraepithelial neoplasia (CIN)2 +  [6]. It has also been recently reported that HPV-35 is one of the most dominant types among South African women with CIN3, only behind HPV-16 [7]. Another study showed that the most common carcinogenic HPV subtypes are HPV-16, HPV-58, and HPV-33 in southwest China [8]. Therefore, in addition to HPV-16 and HPV-18, other putative HPV carcinogenic types warrant our attention.

There has been an increasing trend in recent years towards multiple HR-HPV infections [9]; however, the clinical significance of multiple HPV infections is controversial [10]. Some studies have shown that multiple HPV infections lead to an increased risk of cervical lesions compared to single HPV infections [11, 12]; however, other studies have shown that compared to single HR-HPV infections, multiple HR-HPV infections do not increase the risk of cervical cancer [13, 14]. Furthermore, whether there are differences between HR-HPV genotypes and whether a specific combination of HR-HPV genotypes will increase or reduce the risk of cervical cancer warrants further study.

In the current study, we determined the prevalence of HPV and the genotype-specific distribution in cervical cytologic abnormalities in Chongqing, China. Furthermore, the correlation between multiple HR-HPV infections and cervical pathological abnormalities (including TCT and biopsies of colposcopy) was also determined. The type-type interactions of multiple HPV infections on cervical disease risk were investigated. The aim of this study was to provide an in-depth assessment of the prevalence of multiple HR-HPV infections.

## Material and methods

### Study design and setting

#### Clinical specimen collection

**Fig. 1:**
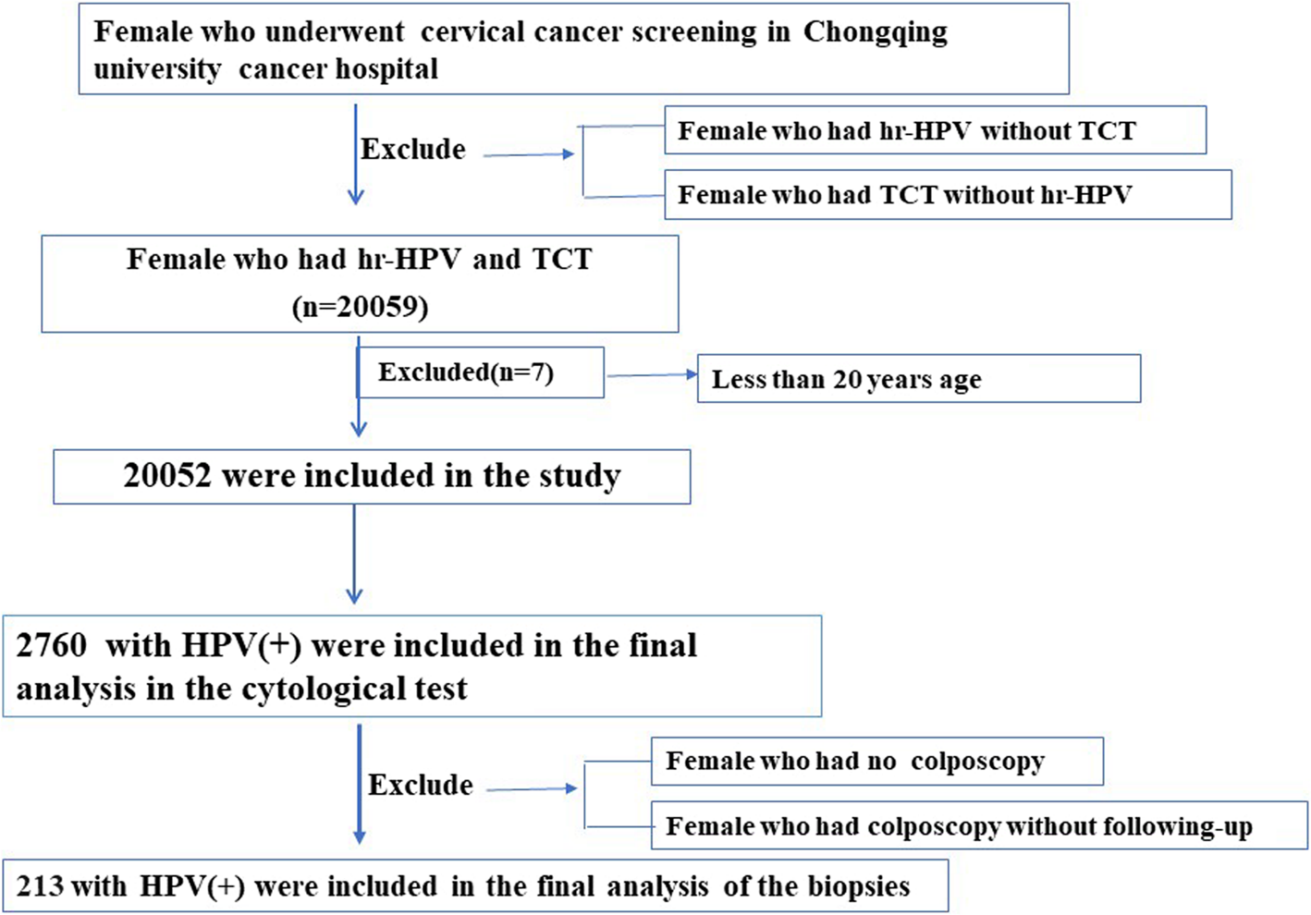
Flow chart of study participants. TCT = Thinprep cytologic test

This study included 20,059 women who underwent physical examinations between January 2015 and December 2021 at the Chongqing University Cancer Hospital (Chongqing, China). The age of the patients ranged from 16–86 years. All patients underwent HPV genotype testing and cytologic screening (TCT). At the subsequent follow-up evaluation, there were 144 and 69 patients with single and multiple infections, respectively, who underwent colposcopy because of HPV-positivity or abnormal cytology. The screening flowchart is shown in Fig. 1. Participants provided written informed consent, and procedures were approved by the Ethics Committee of Chongqing University Cancer Hospital.

### Specimen collection and testing

Clinical examinations were performed on participants. Cervical cells were obtained using a cytobrush, which was then stored in cytosol. HPV testing was done on exfoliated cervical cells in the Department of Pathology. DNA was extracted from the samples, followed by PCR and HPV genotyping. according to the manufacturer’s instructions (China Shanghai ZJ Bio-Tech Co., Ltd). Fifteen HR-HPV genotypes (oncogenic; HPV-16, -18, -31, -33, -35, -39, -45, -51, -52, -56, -58, -59, -66, -68, and -82) were detected. The PCR program consisted with the following parameters: 94˚C for 2 min, then 40 cycles at 93 ˚C for 10s, 62 ˚C for 31s.

### Cytologic and pathologic diagnoses

Classifications of lesions in TCT were performed in conformity with the Bethesda 2015 criteria [15], including negative for intraepithelial lesions or malignancies (NILMs), which includes normal and inflammatory tissues, atypical squamous cells of undetermined significance/cannot exclude high-grade lesion (ASC-US), low-grade squamous intraepithelial lesions (LSILs), high-grade squamous intraepithelial lesions (HSILs), cervical squamous cell carcinoma (SCC), and atypical glandular cells (AGCs). The biopsy specimens obtained were fixed in formalin, embedded in paraffin, and stained with hematoxylin–eosin according to a standard protocol. The histologic diagnosis was established using standard criteria and cervical intraepithelial neoplasia (CIN) terminology.

## Statistical analysis

Data were analyzed using IBM SPSS Statistics (version 23.0). A chi-squared test or Fisher's exact test was used to compare categorical variables. Multivariate logistic regression was used to determine the association between each pairing of HPV types in ASCUS, LSILs, and HSILs. Furthermore, additive statistical interactions of type-type on risk of ASCUS, LSILs, and HSILs were assessed by computing synergy indices and 95% confidence intervals [16]. The synergy index was calculated as follows: [exp(b3)-1]/[(exp(b1) + exp(b2)-2]. For example, for HPV-31and HPV-33, where b1 is the main effect of HPV-31, b2 is the main effect of HPV-33, and b3 is the coefficient for the cross-product term between HPV-31 andHPV-33 in a logistic regression model.

## Results

In the present survey, 19.49% of the HPV-positive samples had multiple HR-HPV infections. All 20,059 samples were divided into 10 age groups (≤ 20 years, 21–25 years, 26–30 years, 31–35 years, 36–40 years, 41–45 years, 46–50 years, 51–55 years, 56–60 years, and > 60 years). There were only 7 people in the ≤ 20 years age group, thus they were not included in the data analysis. Of the HPV-positive samples, 19.49% (538/2760) had multiple HR-HPV infections. The HPV overall infection prevalence in different age groups (21–25 years, 26–30 years, 31–35 years, 36–40 years, 41–45 years, 46–50 years, 51–55 years, 56–60 years, and > 60 years) was 13.41%, 13.45%, 12.82%, 11.60%, 12.30%, 12.72%, 15.03%, 17.69%, and 23.00%, respectively. The overall HPV-positive rate was highest in the > 60 years age group, followed by the 56–60 and 51–55 years age groups. The HPV multiple infection prevalence in different ages (21–25 years, 26–30 years, 31–35 years, 36–40 years, 41–45 years, 46–50 years, 51–55 years, 56–60 years, and > 60 years) was 4.62%, 2.42%, 2.41%, 1.67%, 1.72%, 2.67%, 2.76%, 3.95%, and 7.14%, respectively. There was a typical bimodal pattern among multiple HPV infections, with a peak in the younger group and a second peak in the elderly group. A bimodal pattern was not present in the prevalence of single HR-HPV infections (Table 1).

**Table 1:** Prevalence of total, multiple, and single HPV infections at different ages

Age (years)	Total *n* = 20052	HPV positive, n (%) *n* = 2760	Single infection, n (%) *n* = 2222	Multiple infection, n (%) *n* = 538
21–25	455	61(13.41%)	40(8.79%)	21(4.62%)
26–30	2104	283(13.45%)	232(11.03%)	51(2.42%)
31–35	3199	410(12.82%)	333(10.41%)	77(2.41%)
36–40	2810	326(11.60%)	279(9.93%)	47(1.67%)
41–45	2739	337(12.30%)	290(10.59%)	47(1.72%)
46–50	3521	448(12.72%)	354(10.05%)	94(2.67%)
51–55	2968	446(15.03%)	364(12.26%)	82(2.76%)
56–60	1317	233(17.69%)	181(13.74%)	52(3.95%)
> 60	939	216(23.00%)	149(15.87%)	67(7.14%)

HPV-52, HPV-58, HPV-16, HPV-51, and HPV-39 were the most common HR-HPV genotypes, accounting for 4.74% (951/20,052), 2.13% (427/20,052), 1.98% (397/20,052), 1.22% (245/20,052), and 1.17% (235/20,052) of HR-HPV infections, respectively.

For each individual HPV age trend, HPV-16, HPV-39, HPV-51, HPV-56, and HPV-66 exhibited a bimodal pattern. These genotypes were increased in the 21–25 and 26–30 year age groups, then began to decline and increased again in the 51–55 year age group, with a peak in the > 60 year age group. The other HR-HPV genotypes did not exhibit a bimodal trend (Table 2).

**Table 2:** Age-related overall prevalence of each HPV genotype

Genotypes	Age(years),n (%)								
	**21–25**	**26–30**	**31–35**	**36–40**	**41–45**	**46–50**	**51–55**	**56–60**	** > 60**
	***n* = 455**	***n* = 2104**	***n* = 3199**	***n* = 2810**	***n* = 2739**	***n* = 3521**	***n* = 2968**	***n* = 1317**	***n* = 939**
HPV-16	13(2.86%)	51(2.42%)	60(1.88%)	53(1.89%)	44(1.61%)	61(1.73%)	61(2.06%)	28(2.13%)	26(2.77%)
HPV-18	3(0.66%)	17(0.81%)	21(0.66%)	13(0.46%)	18(0.66%)	24(0.68%)	30(1.01%)	7(0.53%)	13(1.38%)
HPV-31	2(0.44%)	12(0.57%)	16(0.50%)	13(0.46%)	9(0.33%)	16(0.45%)	9(0.30%)	11(0.84%)	9(0.96%)
HPV-33	1(0.22%)	8(0.38%)	7(0.22%)	16(0.57%)	13(0.47%)	21(0.60%)	12(0.40%)	12(0.91%)	11(1.17%)
HPV-35	1(0.22%)	8(0.38%)	9(0.28%)	3(0.11%)	7(0.26%)	17(0.48%)	12(0.40%)	12(0.91%)	8(0.85%)
HPV-39	9(1.98%)	23(1.09%)	37(1.16%)	26(0.93%)	26(0.95%)	36(1.02%)	37(1.25%)	22(1.67%)	19(2.02%)
HPV-45	1(0.22%)	4(0.19%)	5(0.16%)	3(0.11%)	2(0.07%)	10(0.28%)	6(0.20%)	3(0.23%)	5(0.53%)
HPV-51	14(3.08%)	31(1.47%)	41(1.28%)	30(1.07%)	24(0.88%)	35(0.99%)	32(1.08%)	16(1.21%)	22(2.34%)
HPV-52	21(4.62%)	96(4.56%)	141(4.41%)	112(3.99%)	116(4.24%)	146(4.15%)	143(4.82%)	95(7.21%)	81(8.63%)
HPV-56	9(1.98%)	22(1.05%)	18(0.56%)	18(0.64%)	28(1.02%)	33(0.94%)	35(1.18%)	18(1.37%)	19(2.02%)
HPV-58	7(1.54%)	29(1.38%)	65(2.03%)	45(1.60%)	45(1.64%)	77(2.19%)	82(2.76%)	36(2.73%)	41(4.37%)
HPV-59	6(1.32%)	13(0.62%)	25(0.78%)	15(0.53%)	17(0.62%)	25(0.71%)	23(0.78%)	7(0.53%)	7(0.75%)
HPV-66	7(1.54%)	27(1.28%)	16(0.50%)	20(0.71%)	21(0.77%)	31(0.88%)	33(1.11%)	14(1.06%)	20(2.13%)
HPV-68	4(0.88%)	14(0.67%)	27(0.84%)	10(0.36%)	23(0.84%)	27(0.77%)	36(1.21%)	15(1.14%)	18(1.92%)
HPV-82	0(0.00%)	5(0.24%)	6(0.19%)	6(0.21%)	3(0.11%)	9(0.26%)	4(0.13%)	4(0.30%)	4(0.43%)

HPV-52, HPV-58, HPV-16, HPV-39, and HPV-56 were the most common multiple infections, accounting for 1.25%, 0.75%, 0.63%, 0.49%, and 0.48%, respectively. Most multiple HPV genotypes exhibited a bimodal pattern, except HPV-33 and HPV-82 (Table 3).

**Table 3:** Multiple HPV genotype infections according to different ages

	**Age(years), n (%)**								
Genotypes	**21–25**	**26–30**	**31–35**	**36–40**	**41–45**	**46–50**	**51–55**	**56–60**	** > 60**
HPV-16	7(1.54%)	13(0.62%)	17(0.53%)	14(0.50%)	12(0.44%)	26(0.74%)	17(0.57%)	9(0.68%)	12(1.28%)
HPV-18	2(0.44%)	6(0.29%)	8(0.25%)	4(0.14%)	8(0.29%)	10(0.28%)	8(0.27%)	5(0.38%)	5(0.53%)
HPV-31	1(0.22%)	5(0.24%)	7(0.22%)	2(0.07%)	3(0.11%)	4(0.11%)	2(0.07%)	6(0.46%)	5(0.53%)
HPV-33	0(0.00%)	2(0.10%)	2(0.06%)	5(0.18%)	1(0.04%)	6(0.17%)	2(0.07%)	8(0.61%)	5(0.53%)
HPV-35	1(0.22%)	2(0.10%)	2(0.06%)	1(0.04%)	2(0.07%)	5(0.14%)	4(0.13%)	7(0.53%)	5(0.53%)
HPV-39	7(1.54%)	12(0.57%)	19(0.59%)	7(0.25%)	7(0.26%)	10(0.28%)	15(0.51%)	9(0.68%)	12(1.28%)
HPV-45	1(0.22%)	2(0.10%)	2(0.06%)	2(0.07%)	2(0.07%)	4(0.11%)	1(0.03%)	2(0.15%)	3(0.32%)
HPV-51	10(2.20%)	8(0.38%)	12(0.38%)	9(0.32%)	6(0.22%)	15(0.43%)	10(0.34%)	7(0.53%)	16(1.70%)
HPV-52	9(1.98%)	29(1.38%)	36(1.13%)	21(0.75%)	20(0.73%)	47(1.33%)	36(1.21%)	22(1.67%)	31(3.30%)
HPV-56	5(1.10%)	11(0.52%)	9(0.28%)	5(0.18%)	9(0.33%)	19(0.54%)	16(0.54%)	10(0.76%)	13(1.38%)
HPV-58	4(0.88%)	11(0.52%)	17(0.53%)	10(0.36%)	15(0.55%)	30(0.85%)	28(0.94%)	14(1.06%)	22(2.34%)
HPV-59	3(0.66%)	5(0.24%)	11(0.34%)	4(0.14%)	5(0.18%)	10(0.28%)	11(0.37%)	3(0.23%)	5(0.53%)
HPV-66	4(0.88%)	12(0.57%)	8(0.25%)	12(0.43%)	5(0.18%)	14(0.40%)	14(0.47%)	9(0.68%)	11(1.17%)
HPV-68	3(0.66%)	3(0.14%)	14(0.44%)	5(0.18%)	7(0.26%)	14(0.40%)	18(0.61%)	6(0.46%)	10(1.06%)
HPV-82	0(0.00%)	4(0.19%)	3(0.09%)	3(0.11%)	0(0.00%)	4(0.11%)	1(0.03%)	3(0.23%)	4(0.43%)

Among all HR-HPV genotypes, each HR-HPV genotype was more frequently detected in patients with multiple HPV infections than single HPV infections (Table 4; *p* < 0.05).

**Table 4:** Distribution of HPV genotypes (single and multiple infections)

Genotypes	Single infection, n (%) *n* = 2222	Multiple infection, n (%) *n* = 538
HPV-16	270(12.15%)	127(23.61%)
HPV-18	90(4.05%)	56(10.41%)
HPV-31	62(2.79%)	35(6.51%)
HPV-33	70(3.15%)	31(5.76%)
HPV-35	48(2.16%)	29(5.39%)
HPV-39	137(6.17%)	98(18.22%)
HPV-45	20(0.90%)	19(3.53%)
HPV-51	152(6.84%)	93(17.29%)
HPV-52	700(31.50%)	251(46.65%)
HPV-56	103(4.64%)	97(18.03%)
HPV-58	276(12.42%)	151(28.07%)
HPV-59	81(3.65%)	57(10.59%)
HPV-66	100(4.50%)	89(16.54%)
HPV-68	94(4.23%)	80(14.87%)
HPV-82	19(0.86%)	22(4.09%)

The correlations between overall single/multiple HPV infections and different cervical lesions were analyzed. There were 2222 cases of HPV single‐type infections grades as follows: normal, 87.58% (1946/2222); ASCUS, 6.17% (137/2222); LSIL, 4.14% (92/2222); and HSIL, 1.94% (43/2222). Of the 538 patients with multiple HPV genotype infections, the grades were as follows: normal, 80.11% (431/538); ASCUC, 7.25% (39/538); LSIL, 7.81% (42/538); and HSIL, 4.28% (23/538).

NMIL was more frequent in single HPV infections than HPV multiple infections. HSIL and LSIL were more frequent in multiple HPV infections than single HPV infections (*p* < 0.05). ASCUS was also more frequently detected in multiple HPV infections (37/236 [15.7%]) than single HPV infections, although the difference was not statistically significant (Table 5).

**Table 5:** Correlation between TCT and HPV infection status (single and multiple)

**TCT**	**HPV Infection Status**		
	**Single Infection, n (%)** ***n* = 2222**	**Multiple Infection, n (%)** ***n* = 538**	***P* value**
Normal	1946 (87.58%)^b^	431 (80.11%)^a^	< 0.001
ASCUS	137 (6.17%)^a^	39 (7.25%)^a^	
LSIL	92 (4.14%)^b^	42 (7.81%)^a^	
HSIL	43 (1.94%)^b^	23 (4.28%)^a^	

Different letters indicate statistically significant differences in the proportion of multiple and single HPV infections (*P* < 0.05)

The most common HPV type in patients with HSIL was HPV-16, followed by HPV-52, HPV-58, and HPV-33. Among patients with LSIL, HPV-52 was the most common type, followed by HPV-58, HPV-66, and HPV51. Among patients with ASCUS, HPV-52, followed by HPV-58, HPV-16, and HPV-68 were the most common HPV types. The most common multiple HPV types in patients with HSIL was HPV-16 (56.52%), HPV-52 (47.83%), HPV-58 (21.74%), HPV-33 (17.39%), and HPV-66 (17.39%). The most common single HPV types in patients with HSIL was HPV-16 (37.21%), HPV-52 (23.26%), HPV-58 (13.95%), HPV-33 (13.95%). The cell abnormalities caused by HPV-45 and HPV-82 were lower than the cell abnormalities caused by other genotypes, whether a single or multiple infection. Only one patient with a single HPV infection and ASCUS was caused by HPV-45, and only one patient with multiple HPV infections and LSIL was caused by HPV-82 (Table 6).

**Table 6:** Association between TCT and HPV genotypes (single and multiple HPV infections)

Genotypes	**Normal**		**ASCUS**		**LSIL**		**HSIL**		**AGC**		**S CC**	
	**Single**	**Multiple**	**Single**	**Multiple**	**Single**	**Multiple**	**Single**	**Multiple**	**Single**	**Multiple**	**Single**	**Multiple**
	***n* = 1946**	***n* = 431**	***n* = 137**	***n* = 39**	***n* = 92**	***n* = 42**	***n* = 43**	***n* = 23**	***n* = 4**	***n* = 2**	***n* = 0**	***n* = 1**
HPV-16	228(11.72%)	97(22.51%)	15(10.95%)	8(20.51%)	10(10.87%)	8(19.05%)	16(37.21%)	13(56.52%)	1(25.00%)	0(0.00%)	0(0.00%)	1(100%)
HPV-18	80(4.11%)	43(9.98%)	7(5.11%)	4(10.26%)	3(3.26%)	6(14.29%)	0(0.00%)	3(13.04%)	0(0.00%)	0(0.00%)	0(0.00%)	0(0.00%)
HPV-31	54(2.77%)	31(7.19%)	4(2.92%)	1(2.56%)	4(4.35%)	2(4.76%)	0(0.00%)	0(0.00%)	0(0.00%)	1(50.00%)	0(0.00%)	0(0.00%)
HPV-33	58(2.98%)	21(4.87%)	3(2.19%)	2(5.13%)	3(3.26%)	4(9.52%)	6(13.95%)	4(17.39%)	0(0.00%)	0(0.00%)	0(0.00%)	0(0.00%)
HPV-35	47(2.42%)	27(6.26%)	0(0.00%)	1(2.56%)	1(1.09%)	0(0.00%)	0(0.00%)	1(4.35%)	0(0.00%)	0(0.00%)	0(0.00%)	0(0.00%)
HPV-39	130(6.68%)	82(19.03%)	3(2.19%)	9(23.08%)	4(4.35%)	4(9.52%)	0(0.00%)	1(4.35%)	0(0.00%)	1(50.00%)	0(0.00%)	1(100%)
HPV-45	19(0.98%)	18(4.18%)	1(0.73%)	1(2.56%)	0(0.00%)	0(0.00%)	0(0.00%)	0(0.00%)	0(0.00%)	0(0.00%)	0(0.00%)	0(0.00%)
HPV-51	127(6.53%)	75(17.40%)	11(8.03%)	8(20.51%)	12(13.04%)	9(21.43%)	2(4.65%)	0(0.00%)	0(0.00%)	0(0.00%)	0(0.00%)	1(100%)
HPV-52	617(31.71%)	195(45.24%)	51(37.23%)	23(58.97%)	19(20.65%)	20(47.62%)	10(23.26%)	11(47.83%)	3(75.00%)	2(100.00%)	0(0.00%)	0(0.00%)
HPV-56	91(4.68%)	80(18.56%)	9(6.57%)	7(17.95%)	3(3.26%)	7(16.67%)	0(0.00%)	3(13.04%)	0(0.00%)	0(0.00%)	0(0.00%)	0(0.00%)
HPV-58	236(12.13%)	123(28.54%)	16(11.68%)	11(28.21%)	18(19.57%)	12(28.57%)	6(13.95%)	5(21.74%)	0(0.00%)	0(0.00%)	0(0.00%)	0(0.00%)
HPV-59	74(3.80%)	50(11.60%)	4(2.92%)	3(7.69%)	3(3.26%)	1(2.38%)	0(0.00%)	2(8.70%)	0(0.00%)	0(0.00%)	0(0.00%)	1(100%)
HPV-66	87(4.47%)	67(15.55%)	4(2.92%)	4(10.26%)	7(7.61%)	14(33.33%)	2(4.65%)	4(17.39%)	0(0.00%)	0(0.00%)	0(0.00%)	0(0.00%)
HPV-68	79(4.06%)	61(14.15%)	9(6.57%)	12(30.77%)	5(5.44%)	6(14.29%)	1(2.33%)	1(4.35%)	0(0.00%)	0(0.00%)	0(0.00%)	0(0.00%)
HPV-82	19(0.98%)	21(4.87%)	0(0.00%)	0(0.00%)	0(0.00%)	1(2.38%)	0(0.00%)	0(0.00%)	0(0.00%)	0(0.00%)	0(0.00%)	0(0.00%)

We then performed follow-up evaluations of patients with additional biopsies in our hospital (including 144 single and 69 multiple infections). In the single HPV infection group, 4, 3, 2, and 2 of the 12 patients with cervical cancer were positive for HPV-16, HPV-58, HPV-33 and HPV-18, respectively. HPV-16 was also the most prevalent genotype among patients with cervical cancer and multiple HPV infections, followed by HPV-58, HPV-52, and HPV-18 (Table 7).

**Table 7:** The number of HPV subtypes in different pathologic grades of cervical lesions

Genotypes	Chronic cervicitis		CIN1-CIN2		CIN3		Cervical cancer	
	Single	Multiple	Single	Multiple	Single	Multiple	Single	Multiple
	*n* = 56	*n* = 15	*n* = 45	*n* = 22	*n* = 31	*n* = 20	*n* = 12	*n* = 12
HPV16	33(58.93%)	10(66.67%)	17(37.78%)	6(27.27%)	23(74.19%)	13(65.00%)	4(33.33%)	9(75.00%)
HPV18	11(19.64)	9(60.00%)	4(8.89%)	8(36.36%)	1(3.23%)	2(10.00%)	2(16.67%)	2(16.67%)
HPV31	0(0.00%)	1(6.67%)	1(2.22%)	1(4.55%)	0(0.00%)	0(0.00%)	0(0.00%)	0(0.00%)
HPV33	0(0.00%)	0(0.00%)	0(0.00%)	0(0.00%)	1(3.23%)	3(15.00%)	2(16.67%)	0(0.00%)
HPV35	0(0.00%)	1(6.67%)	0(0.00%)	1(4.55%)	0(0.00%)	0(0.00%)	0(0.00%)	0(0.00%)
HPV39	0(0.00%)	0(0.00%)	0(0.00%)	1(4.55%)	0(0.00%)	4(20.00%)	0(0.00%)	1(8.33%)
HPV45	0(0.00%)	0(0.00%)	0(0.00%)	0(0.00%)	0(0.00%)	0(0.00%)	0(0.00%)	0(0.00%)
HPV51	0(0.00%)	5(33.33%)	6(13.33%)	2(9.09%)	1(3.23%)	2(10.00%)	0(0.00%)	1(8.33%)
HPV52	3(5.36%)	13(86.67%)	8(17.78%)	8(36.36%)	1(3.23%)	9(45.00%)	0(0.00%)	4(33.33%)
HPV56	1(1.79%)	5(33.33%)	1(2.22%)	2(9.09%)	1(3.23%)	3(15.00%)	0(0.00%)	1(8.33%)
HPV58	1(1.79%)	4(26.67%)	4(8.89%)	4(18.18%)	2(6.45%)	3(15.00%)	4(33.33%)	5(41.67%)
HPV59	0(0.00%)	1(6.67%)	2(4.44%)	0(0.00%)	0(0.00%)	1(5.00%)	0(0.00%)	1(8.33%)
HPV66	0(0.00%)	2(13.33%)	0(0.00%)	6(27.27%)	1(3.23%)	1(5.00%)	0(0.00%)	1(8.33%)
HPV68	3(5.36%)	2(13.33%)	1(2.22%)	3(13.64%)	0(0.00%)	2(10.00%)	0(0.00%)	0(0.00%)
HPV82	4(7.14%)	1(6.67%)	1(2.22%)	2(9.09%)	0(0.00%)	0(0.00%)	0(0.00%)	0(0.00%)

Among the different cervical pathologic grades, patients with cervical cancer had the higher percentage of multiple HPV infections (50%) compared to chronic cervicitis (21.13%) and the difference was statistically significant (*P* < 0.05). The multiple HPV infections in CIN3 (36.22%), CIN1-CIN2 (32.84%) were both lower that in cervical cancer, higher that in chronic cervicitis, although the difference was not statistically significant (Table 8).

**Table 8:** Analysis of single and multiple HPV infections and cervical pathologic grade

	**Total (NO.)**	**Multiple infections**	**Single infections**	***P***
Chronic cervicitis	71	15(21.13%)^a^	56(78.87%)^a^	0.035
CIN1-CIN2	67	22(32.84%)^ab^	45(67.16%)^ab^	
CIN3	51	20(39.22%)^ab^	31(60.78%)^ab^	
Cervical cancer	24	12(50.00%)^b^	12(50.00%)^b^	

Different letters indicate statistical differences in the proportion of multiple HPV and single HPV infections (*P* < 0.05). Chronic cervicitis, CIN1, CINII correspond to a, ab, ab, all have the letter a, and the three are not statistically significant. The letters corresponding to chronic inflammation and cervical cancer were a and b, respectively, which were statistically significant

**Fig. 2:**
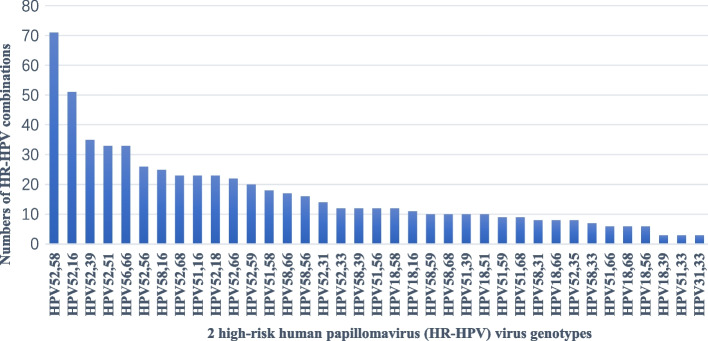
Distribution involving 2 high-risk human papillomavirus (HR-HPV) virus genotypes in patients with multiple infections

In the current study the most common HPV genotype combinations were HPV-52 + HPV-58 (71 cases) and HPV-52 + HPV-16 (51 cases). The other common multiple HPV infections were HPV-52 + HPV-39 (35 cases), HPV-66 + HPV-56 (33 cases) and HPV-51 + HPV-52 (33 cases) (Fig. 2).

Women with multiple infections were at increased risk of LSIL (OR, 2.01; 95% CI, 1.38–2.93) and HSIL (OR, 2.28; 95% CI,1.36–3.81) when compared with single infections. The odds ratios (ORs) and 95% confidence intervals (CIs) in patients with abnormal cytology and multiple infections were calculated for each HPV genotype and compared with single infections. For HPV-52, co-infection with additional HR-HPV types increased the HSIL (OR, 3.18; 95% CI, 1.33–7.58) and LSIL risk (OR, 3.11; 95% CI, 1.63–5.94) when compared to single infections. For HPV-39, co-infection with additional HR-HPV types increased ASCUS risk (OR, 4.18; 95% CI, 1.08–16.08) when compared with single infections. For each HPV types (58, 16, 33, 66, 68) co-infection with additional HR-HPV types marginally increased compared with single infections (Table 9).

**Table 9:** Relationship between single and multiple HR-HPV infection and risk of cervical disease

	NO	ASCUS		LSIL		HSIL	
		n (*%*)	**OR**(95%*CI*)	n (*%*)	**OR(**95%*CI*)	(*%*)	**OR**(95%*CI*)
Single	2222	137(6.2)	1.00	92(4.1)	1.00	43(1.9)	1.00
Multiple-	538	39(7.2)	1.21(0.83–1.75)	42(7.8)	2.01(1.38–2.93)	23(4.3)	2.28(1.36–3.81)
HPV82							
Single	19	0(0.0)	N/A	0(0.0)	N/A	0(0.0)	N/A
Multiple	22	0(0.0)		1(4.5)		0(0.0)	
HPV45							
Single	20	1(5.0)	1.00	0(0.0)	N/A	0(0.0)	N/A
Multiple	19	1(5.3)	0.70(0.03–14.63)	0(0.0)		0(0.0)	
HPV35							
Single	48	0(0.0)	N/A	1(2.1)	N/A	0(0.0)	N/A
Multiple	29	1(3.4)		0(0.0)		1(3.4)	
HPV33							
Single	70	3(4.3)	1.00	3(4.3)	1.00	6(8.6)	1.00
Multiple	31	2(6.5)	1.81(0.28–11.88)	4(12.9)	3.87(0.78–19.26)	4(12.9)	2.12(0.52–8.67)
HPV31							
Single	62	4(6.5)	1.00	4(6.5)	1.00	0(0.0)	N/A
Multiple	35	1(2.9)	0.37(0.04–3.64)	2(5.7)	0.88(0.15–5.08)	0(0.0)	
HPV59							
Single	81	4(4.9)	1.00	3(3.7)	1.00	0(0.0)	N/A
Multiple	57	3(5.3)	1.10(0.24–5.15)	1(1.8)	0.46(0.05–4.58)	2(3.5)	
HPV18							
Single	90	7(7.8)	1.00	3(3.3)	1.00	0(0.0)	N/A
Multiple	56	4(7.1)	0.90(0.25–3.26)	6(10.7)	3.51(0.83–14.89)	3(5.4)	
HPV68							
Single	94	9(9.6)	1.00	5(5.3)	1.00	1(1.1)	1.00
Multiple	80	12(15.0)	1.71(0.68–4.33)	6(7.5)	1.52(0.44–5.21)	1(1.3)	1.23(0.08–20.17)
HPV66							
Single	100	4(4.0)	1.00	7(7.0)	1.00	2(2.0)	1.00
Multiple	89	4(4.5)	1.14(0.28–4.69)	14(15.7)	2.50(0.96–6.51)	4(4.5)	2.33(0.42–13.05)
HPV56							
Single	103	9(8.7)	1.00	3(2.9)	1.00	0(0.0)	N/A
Multiple	97	7(7.2)	0.83(0.30–2.34)	7(7.2)	2.65(0.66–10.61)	3(3.1)	
HPV39							
Single	137	3(2.2)	1.00	4(2.9)	1.00	0(0.0)	N/A
Multiple	98	9(9.2)	4.18(1.08–16.08)	4(4.1)	1.37(0.33–5.68)	1(1.0)	
PV51							
Single	152	11(7.2)	1.00	12(7.9)	1.00	2(1.3)	N/A
Multiple	93	8(8.6)	1.28(0.49–3.33)	9(9.7)	1.31(0.53–3.26)	0(0.0)	
HPV16							
Single	270	15(5.6)	1.00	10(3.7)	1.00	16(5.9)	1.00
Multiple	127	8(6.3)	1.13(0.47–2.74)	8(6.3)	1.78(0.69–4.64)	13(10.2)	1.76(0.82–3.79)
HPV58							
Single	276	16(5.8)	1.00	18(6.5)	1.00	6(2.2)	1.00
Multiple	151	11(7.3)	1.32(0.59–2.94)	12(7.9)	1.32(0.62–2.84)	5(3.3)	1.51(0.45–5.08)
HPV52							
Single	700	51(7.3)	1.00	19(2.7)	1.00	10(1.4)	1.00
Multiple	251	23(9.2)	1.30(0.78–2.18)	20(8.0)	3.11(1.63–5.94)	11(4.4)	3.18(1.33–7.58)

Odds ratios were adjusted according to age

*N/A* Not applicable

Under normal conditions, the OR of single infections was higher than multiple infections. In the current study the most common multiple infections were a9 genotypes 16, 31, 33, 35, 58,66, 56, 51, and 52). Therefore, we evaluated evidence for type-type interactions on cervical disease risk (Table 10). Several genotypes acted in combination to increase the risk of HSIL. The synergy indices of HPV-33 and HPV-16 (OR, 2.08; 95% CI, 1.38–3.14), and HPV-33 and HPV-52 (OR, 4.56; 95% CI, 2.60–8.04) were > 1.0 in HSIL. The majority of other synergy indices were not estimated. There may be evidence for an HPV-33 and HPV-31interaction in increasing LSIL risk. The synergy index was 47.14(95% CI, 10.51–211.48) in LSIL. In addition, the synergy index did not increase significantly in the interaction of other a9 genotypes for HSIL and LSIL (Table 10).

**Table 10:** Interaction between co-infection of genotypes, HPV-66 and HPV-56, and risk of cervical disease

Variable	ASCUS[**OR (**95%*CI*)]	LSIL[**OR** (95%*CI*)]	HSIL[**OR** (95%*CI*)]
HPV35-HPV33			
Negative	1.00	1.00	1.00
HPV35	0.19(0.03–1.37)	0.26(0.04–1.89)	0.61(0.08–4.49)
HPV33	0.75(0.30–1.86)	1.47(0.67–3.23)	5.11(2.52–10.34)
HPV35 and HPV33	NE	NE	NE
Synergy index	NE	NE	NE
HPV35-HPV31			
Negative	1.00	1.00	1.00
HPV35	0.19(0.03–1.37)	0.26(0.04–1.87)	0.52(0.07–3.78)
HPV31	0.78(0.31–1.95)	1.29(0.55–3.01)	NE
HPV35 and HPV31	NE	NE	NE
Synergy index	NE	NE	NE
HPV35-HPV16			
Negative	1.00	1.00	1.00
HPV35	0.20(0.03–1.42)	0.26(0.04–1.91)	0.87(0.12–6.43)
HPV16	0.87(0.56–1.37)	0.91(0.55–1.51)	4.99(3.02–8.24)
HPV35 and HPV16	NE	NE	NE
Synergy index	NE	NE	NE
HPV35-HPV58			
Negative	1.00	1.00	1.00
HPV35	0.21(0.03–1.52)	0.31(0.04–2.24)	0.60(0.08–4.41)
HPV58	0.98(0.64–1.50)	1.62(1.06–2.46)	1.10(0.57–2.13)
HPV35 and HPV58	NE	NE	NE
Synergy index	NE	NE	NE
HPV35-HPV52			
Negative	1.00	1.00	1.00
HPV35	NE	0.26(0.04–1.87)	0.57(0.08–4.18)
HPV52	1.35(0.99–1.84)	0.76(0.52–1.11)	0.88(0.52–1.49)
HPV35 and HPV52	2.29(0.28–18.82)	NE	NE
Synergy index	NE	NE	NE
HPV33-HPV31			
Negative	1.00	1.00	1.00
HPV33	0.77(0.31–1.92)	1.29(0.55–3.01)	5.03(2.49–10.19)
HPV31	0.80(0.32–2.00)	1.11(0.44–2.79)	NE
HPV33 and HPV31	NE	20.02(1.24–321.92)	NE
Synergy index	NE	47.14(10.51–211.48)	NE
HPV33-HPV16			
Negative	1.00	1.00	1.00
HPV33	0.78(0.31–1.94)	1.29(0.55–3.02)	8.17(3.74–17.84)
HPV16	0.89(0.57–1.40)	0.89(0.53–1.49)	6.13(3.59–10.47)
HPV33 and HPV16	NE	6.53(0.67–63.32)	26.64(2.69–264.06)
Synergy index	NE	31.00(0.50–1923.54)	2.08(1.38–3.14)
HPV33-HPV58			
Negative	1.00	1.00	1.00
HPV33	0.64(0.23–1.77)	1.78(0.80–3.94)	5.80(2.83–11.91)
HPV58	0.95(0.62–1.47)	1.70(1.11–2.59)	1.31(0.67–2.56)
HPV33 and HPV58	2.41(0.29–20.13)	NE	NE
Synergy index	-3.47	NE	NE
HPV33-HPV52			
Negative	1.00	1.00	1.00
HPV33	0.79(0.28–2.19)	1.09(0.43–2.76)	3.83(1.66–8.83)
HPV52	1.40(1.02–1.91)	0.74(0.50–1.10)	0.87(0.49–1.53)
HPV33 and HPV52	1.38(0.18–10.72)	3.29(0.72–15.09)	13.29(3.52–50.22)
Synergy index	2.04(0.00–2175.48)	-13.98	4.56(2.60–8.01)
HPV31-HPV16			
Negative	1.00	1.00	1.00
HPV31	0.83(0.33–2.09)	1.39(0.59–3.25)	NE
HPV16	0.90(0.57–1.41)	0.95(0.57–1.58)	4.84(2.94–7.97)
HPV31 and HPV16	NE	NE	NE
Synergy index	NE	NE	NE
HPV31-HPV58			
Negative	1.00	1.00	1.00
HPV31	0.68(0.25–1.88)	1.58(0.67–3.71)	NE
HPV58	0.96(0.62–1.47)	1.69(1.11–2.58)	1.07(0.56–2.07)
HPV31and HPV58	2.07(0.25–16.92)	NE	NE
Synergy index	-2.96	NE	NE
HPV31-HPV52			
Negative	1.00	1.00	1.00
HPV31	1.09(0.43–2.76)	1.18(0.47–2.99)	NE
HPV52	1.44(1.05–1.97)	0.77(0.52–1.13)	0.86(0.51–1.45)
HPV31and HPV52	NE	1.30(0.17–9.99)	NE
Synergy index	NE	-6.08	NE
HPV16-HPV58			
Negative	1.00	1.00	1.00
HPV16	0.96(0.61–1.52)	1.03(0.61–1.76)	5.40(3.15–9.28)
HPV58	1.05(0.68–1.61)	1.67(1.08–2.59)	1.58(0.74–3.38)
HPV16 and HPV58	NE	0.90(0.12–6.71)	6.00(1.35–26.70)
Synergy index	NE	-0.15	1.00(0.47–2.16)
HPV16-HPV52			
Negative	1.00	1.00	1.00
HPV16	1.03(0.62–1.71)	0.78(0.45–1.38)	5.12(2.81–9.31)
HPV52	1.44(1.04–2.00)	0.72(0.48–1.08)	1.24(0.65–2.39)
HPV16 and HPV52	1.05(0.32–3.45)	1.08(0.33–3.54)	7.46(2.70–20.67)
Synergy index	0.11(0.00–57.87)	-0.16	1.48(0.65–3.37)
HPV58-HPV52			
Negative	1.00	1.00	1.00
HPV58	1.00(0.60–1.65)	1.58(0.99–2.52)	1.17(0.57–2.39)
HPV52	1.38(0.99–1.92)	0.83(0.55–1.26)	0.94(0.54–1.64)
HPV58 and HPV52	1.83(0.81–4.12)	1.20(0.42–3.38)	0.58(0.08–4.29)
Synergy index	2.22(0.06–75.95)	0.48(0.01–15.89)	-3.72

Odds ratios were adjusted according to age

*NE* Not estimable

## Discussion

The present study determined the prevalence of HR-HPV genotypes and the correlation with multiple infections and pre-cancer and cancer of the cervix among women in Chongqing, China. In the present survey, 19.49% of the HPV-positive cervical samples had multiple HR-HPV infections. It has been reported that 20%-59% of women are infected with multiple types of HPV [17–19]. The initial HPV studies rarely detected multiple infections, possibly because of the characteristics of early diagnostic tests [20]. The higher prevalence of multiple HPV infections may be due to the increasingly sensitive testing methods now available [21]. The prevalence of multiple infections is affected by diverse factors, including age, socioeconomic status, immune status, and vaccination status [19, 22]. Rousseau [23] concluded that the incidence of multiple HPV types declined markedly with age. Another study also indicated that co-infection with multiple HPV types is more common among younger women [20].

In the current study, the distribution of multiple HPV infections showed a typical “U-shaped” pattern, which is consistent with the findings of a study conducted in Fujian, China [24]. The overall HPV distribution showed a roughly U-shaped pattern, unlike the distribution of single HPV infections. Moreover, the distribution of the multiple HPV infections also showed a “U-shaped” pattern with the exception of HPV-33 and HPV-82. For each HPV type; however, the majority of individual total HPV infections did not exhibit a U-shaped curve. It is possible that the U-shaped curve of multiple infections affects the U-shaped curve of the total infections. Some studies [25, 26] have shown that sexually active women (including women with more sex partners and a higher frequency of sexual intercourse) had the highest risk of multiple infections. It is possible that the increase in multiple infections in postmenopausal women is also due to a decline in immunity [27].

Fifteen HR-HPV genotypes were detected in our study. The five most common HR-HPV genotypes were HPV-52, HPV-58, HPV-16, HPV-51, and HPV-39. The distribution of HPV genotypes varies across different countries, ethnicities, and socioeconomic levels [28].

In Africa, the five most common HR-HPV genotypes, listed in descending order, are HPV-16, HPV-52, HPV-35, HPV-18, and HPV-58, while the most common HR-HPV genotypes in Asia are HPV-16, HPV-52, HPV-58, HPV-33, and HPV-53 [29]. HPV-52, HPV-58, and HPV-16 were also the three most common multiple infections in our study. The most common HPV type in women with HSIL was HPV-16, followed by HPV-52, HPV-58, and HPV-33. A study revealed that the most common HPV types are HPV-16 and HPV-58 among women with HSIL and cervical cancer [30]. Another study showed that persistent HPV-16 and HPV-58 infections are risk factors for cervical disease progression in Korea [31]. Our study also showed that HPV-58 was a common HPV subtype in women with HSIL, CIN3, and cervical cancer, second only to HPV-16. HPV-33 is one of the most common carcinogenic HPV subtypes [32]. Although the prevalence of HPV-33 in the current study was not high, the incidence of HPV-33 in women with HSIL was only less than HPV-16 and HPV-52. Moreover, based on follow-up cervical biopsies, 2 patients with cervical cancer were infected with HPV-33 alone. Adcock [33] also reported that HPV-33 had a low prevalence, but a high positive predictive value (PPV) for precancerous disease and should be managed similar to HPV-16 when detected. Therefore, it can be speculated that HPV-33 and HPV-58 may also be high-risk types in need of increased surveillance and follow-up. Although HPV-52 accounted for the highest proportion of HR-HPV types, most squamous intraepithelial lesions were caused by multiple infections, indicating that single HPV-52 infections were less likely to cause cervical cancer. In the current study the cell abnormalities caused by HPV-45 and HPV-82 were lower than the cellular atypia caused by other genotypes, whether single or multiple infections. HPV-45 was only associated with one case of ASCUS and HSIL caused by single infections, and HPV-82 was only associated by one case of LSIL caused by multiple infections. These results indicate that cervical disease is closely related to HPV type, and the genotype distribution differs regionally.

Many other factors been proven to lead to cervical carcinoma, such as the viral genotype, viral persistence, age, and immune status [34]; however, the clinical importance of multiple HPV types is still controversial compared with single infections. Whether number of infections is a higher risk factor for persistent HPV and cervical lesions remains unclear.

Some studies have reported that the risk of cervical cancer with multiple HPV infections is not higher than single HPV infections [35, 36]. Quint [37] reported that CIN2 and CIN3 are mainly driven by a single HPV type, even if multiple HPV infections are detected. Another study suggested that multiple HPV infections play a role in the occurrence of cervical cancer [38]. In the current study, HSIL and LSIL were more frequent in multiple HPV infections than single HPV infections. The ASCUS group did not have a significantly higher frequency of multiple HPV infections compared to the NILM group. Indeed, NILM had more frequent single HPV infections than multiple HPV infections. Furthermore, based on additional biopsies, single infections occurred more frequently than multiple HPV infections in women with chronic cervicitis; however, multiple HPV infections were more likely to occur in women with cervical cancer.

Whether multiple HPV infections appear randomly or there is a specific combination between HPV types is unknown. A study in Guadeloupe found that the most frequent combinations of HR-HPV were HPV31–33 and HPV31–52 [39]. It has been reported that co-infection with HPV-51 and HPV-52 are also common in the Mexican population [40]. In the current study the most common genotype combinations were HPV-52 and HPV-58 (71 cases), HPV-52 and HPV-16 (51 cases), [HPV-52 and HPV-39] (35 cases), HPV-66 and HPV-56, and HPV-51 and HPV-52 (33cases). HPV52,58,16 are belonging to α9 species.

Laake [41] reported a positive association between HPV-33 and HPV-51. In the current study there were only 3 cases of co-infections between HPV-33 and HPV-51. The combination patterns of each HR-HPV may depend on demographic and a diverse distribution of prevalent genotypes.

To further understand the association between multiple infections and cervical lesions, we further assessed the association between multiple infections and abnormal cytology using logistic regression. Women with multiple infections were at a increased risk of LSIL and HSIL (OR, 2.28; 95% CI, 1.36–3.81) when compared to women with single infections. We further assessed the pathogenicity of each individual genotype in single or multiple infections. For HPV-52, co-infection with additional HR-HPV types increased the HSIL and LSIL risk when compared to single infections. For HPV 39, co-infection with additional HR-HPV types increased the risk for ASCUS. For HPV-58, HPV-16, HPV-33, HPV-66 and HPV-68, co-infection with additional HR-HPV types marginally increased the risk for HSIL when compared to single infections, but the increased risk was not statistically significant.

It is unclear whether there is competition or cooperation among HPV genotypes. It has been suggested that there is no synergistic carcinogenic relationship between specific pairs of HR-HPV types in all grades of cervical neoplasia [42]. In contrast, another study also reported that the specific synergistic interaction between multiple HPVs contributes to cervical cancer [43].

In the current study there may be synergistic carcinogenic relationships between HPV-33 and HPV-16, and HPV-33 and HPV-52 in HSIL, and HPV33-HPV-31 in LSIL. In addition, the synergy index did not increase significantly in the interaction of ɑ9 genotypes in HSIL and LSIL. Some specific combinations synergistically may affect the risk of HSIL and LSIL, but the mechanism underlying these combinations warrants further clinical studies. It is possible that that the diverse distribution of co-infection patterns among multiple HR-HPVs in squamous intraepithelial lesions depends on demographic and other possible risk factors.

This study was limited as a single center study. Many other multicenter studies are needed to confirm the co-infection patterns and mechanism underlying multiple-type infections. In addition, studies including more histologic results are needed.

## Conclusion

In conclusion, we found that HPV-33 and HPV-58 may be HR-HPV types that require increased surveillance and follow-up like HPV-16 and HPV-18. There may be a synergistic carcinogenic relationship between HPV-33 and HPV-16, and HPV-33 and HPV-52 in HSIL, and HPV-33 and HPV-31 in LSIL in our study. There may be some specific combinations that synergistically affected the risk of HSIL and LSIL.

## Data Availability

The datasets used and/or analyzed during the current study are available from the corresponding author on reasonable request.
